# Digital biomarkers derived from wearable sensors in neurorehabilitation: a scoping review

**DOI:** 10.3389/fneur.2026.1908796

**Published:** 2026-09-03

**Authors:** Kholood Matouq Shalabi, Hayam Mahmoud, Shahul Hameed Pakkir Mohamed, Shaffi A. Shaik

**Affiliations:** 1 Department of Rehabilitation Sciences, College of Health and Rehabilitation Sciences, Princess Nourah bint Abdulrahman University, Riyadh, Saudi Arabia; 2 Department of P.T. for Neurological Disorders and its Surgery, Faculty of Physical Therapy, Cairo University, Giza, Egypt; 3 Department of Medical Rehabilitation Sciences, Faculty of Applied Medical Sciences, Umm Al-Qura University, Makkah, Saudi Arabia; 4 Department of Health Rehabilitation Sciences, Faculty of Applied Medical Sciences, University of Tabuk, Tabuk, Saudi Arabia; 5 Saveetha College of Physiotherapy, Saveetha Institute of Medical and Technical Sciences (Deemed to the University), Chennai, Tamil Nadu, India; 6 Family and Community Medicine Department, College of Medicine, King Saud University, Riyadh, Saudi Arabia

**Keywords:** digital biomarkers, inertial measurement units, neurological disorders, neurorehabilitation, Parkinson disease, stroke rehabilitation, wearable sensors

## Abstract

**Background:**

Wearable-derived digital biomarkers are increasingly being investigated in neurological rehabilitation to provide objective and continuous assessment of physical function beyond conventional clinic-based measures. However, the clinical applications and measurement characteristics of these biomarkers across neurological populations remain unclear.

**Objective:**

To synthesize the current evidence regarding wearable-derived digital biomarkers used in neurological rehabilitation and to summarize their clinical applications, biomarker domains, and measurement characteristics across neurological conditions.

**Methods:**

A scoping review was conducted following the PRISMA-ScR framework. Electronic databases including PubMed/MEDLINE, ScienceDirect, Cochrane Library, and Google Scholar were searched for English-language studies published between January 2015 and May 2026. Eligible studies investigated wearable-derived digital biomarkers in neurological rehabilitation populations.

**Results:**

A total of 56 studies were included in the review, with stroke (*n* = 29) and Parkinson disease (*n* = 10) representing the most frequently investigated neurological conditions. Inertial Measurement Unit (IMU) based systems were reported in 34 studies, making them the predominant wearable technology. Kinematic biomarkers were the most commonly investigated biomarker domain, particularly in stroke (16/29) and Parkinson disease studies (9/10). In contrast, activity-based (2/5) and physiological biomarkers (2/5) were more prevalent in spinal cord injury research. Although several studies reported favorable validity and reliability of wearable-derived measures, substantial heterogeneity was observed across studies in sensor configurations, biomarker definitions, and validation methodologies.

**Conclusion:**

Wearable-derived digital biomarkers demonstrate considerable potential for objective and ecologically valid monitoring in neurological rehabilitation. However, methodological heterogeneity and limited longitudinal evidence indicate that clinical implementation remains at an early stage of development.

## 1 Introduction

Neurological disorders are among the leading causes of long-term disability worldwide and are associated with substantial impairments in mobility, balance, upper-limb function, physical activity, and participation in daily life (1). These conditions frequently lead to long-term motor and functional impairments that necessitate extended rehabilitation and continuous monitoring (2). Traditional rehabilitation assessments typically rely on clinical rating scales, observational testing, and intermittent in-clinic evaluations (3). Despite their widespread clinical use and value, these measures may not adequately reflect real-world performance beyond controlled clinical settings (4).

Recent advances in wearable sensors have enabled objective and continuous monitoring of motor performance and rehabilitation outcomes in neurological populations (5). Wearable devices can continuously quantify movement patterns during both structured clinical assessments and free-living activities (6). These technologies offer the potential to overcome limitations associated with subjective clinical scales by providing high-resolution, real-time measures of physical function and activity (5).

A growing body of research has examined wearable-derived digital biomarkers across various neurological conditions (7). These biomarkers have been applied in several rehabilitation contexts, including fall-risk assessment, gait evaluation, rehabilitation monitoring, upper-limb recovery, community mobility assessment, and real-world physical activity monitoring (7, 8). In neurological rehabilitation, wearable-derived biomarkers may provide clinically meaningful information that complements conventional outcome measures. For example, wearable sensors can quantify movement quality and activity patterns, enabling more sensitive detection of functional change over time (8). In stroke rehabilitation, wearable systems have been used to monitor upper-limb use, gait performance, balance, and movement smoothness during real-world activities (9–11). In Parkinson disease (PD), wearable technologies have been applied to quantify gait impairment, postural instability, anticipatory postural adjustments, and freezing of gait (12, 13). Similarly, in other neurological disorders, wearable systems have been investigated for multiple aspects of mobility-related impairments (14–16).

Despite the increasing use of wearable technologies in neurological rehabilitation, several challenges remain regarding the interpretation, standardization, and clinical integration of wearable-derived biomarkers. Substantial heterogeneity exists across studies in several wearable-related factors, including sensor types, placement locations, sampling frequencies, algorithms, outcome measures, validation methods, and intended clinical applications. In addition, while some biomarkers demonstrate strong validity and reliability (17), others show limited clinical value (18). Moreover, previous reviews have mainly focused on specific neurological disorders, individual sensor systems, or isolated rehabilitation applications. Moreover, while previous reviews exist, have mainly focused on specific neurological disorders (19, 20), individual sensor systems (19), or isolated rehabilitation applications (7, 21). Therefore, the clinical applicability of wearable-derived biomarkers across different neurological populations and rehabilitation settings remains unclear. Consequently, there is limited comprehensive synthesis of their broader use in neurological rehabilitation. Therefore, the aim of this review is to systematically map and synthesize the current evidence regarding wearable-derived digital biomarkers used in neurological rehabilitation.

## 2 Methods

This scoping review was conducted following the methodological framework developed by Arksey and O’Malley (22) and later refined by Levac et al. (23). The review was designed and reported in accordance with the Preferred Reporting Items for Systematic Reviews and Meta-Analyses extension for Scoping Reviews (PRISMA-ScR) (24).

### 2.1 Search strategy

A comprehensive literature search was conducted for English-language human studies published between January 2015 and May 2026. The search was performed across PubMed/MEDLINE, ScienceDirect, Cochrane Library, and Google Scholar databases. The search strategy combined terms related to wearable technologies, digital biomarkers, and neurological disorders. In addition, the reference lists of included studies were manually screened to identify potentially relevant articles. The complete search strategy is presented in Supplementary Table S1.

### 2.2 Selection of studies

To refine the study selection criteria, the Population, Concept, and Context (PCC) framework was applied (Table 1).

**Table 1:** Population, concept, and context (PCC) framework used to define the eligibility criteria for study selection.

PCC element	Eligibility criteria
Population	Individuals with neurological disorders undergoing rehabilitation
Concept	Wearable-derived digital biomarkers for assessment, monitoring or validation
Context	Clinical, laboratory, home/community rehabilitation settings

Population: Studies involving adults or children with confirmed neurological disorders were eligible for inclusion. Neurological conditions of interest included stroke, PD, multiple sclerosis, traumatic brain injury, spinal cord injury, cerebral palsy, and other neurological disorders investigated within neurorehabilitation settings. Studies involving healthy controls alone, non-neurological musculoskeletal conditions, or psychiatric disorders without a neurological component were excluded.

Concept: Eligible studies investigated digital biomarkers derived from wearable sensors within four primary domains: kinematic, kinetic, physiological, and activity-based biomarkers. Studies using only non-wearable technologies, such as optical motion capture systems or floor-mounted force plates without wearable integration, were excluded. Studies without extraction or reporting of digital biomarkers, as well as purely qualitative studies, were also excluded.

Context: Studies conducted in inpatient rehabilitation, outpatient rehabilitation, community-based rehabilitation, home monitoring, telerehabilitation, and real-world monitoring settings were eligible for inclusion. Moreover, studies conducted exclusively in highly controlled laboratory environments without clear clinical application or rehabilitation relevance were excluded.

In addition, eligible study designs included randomized controlled trials, non-randomized clinical trials, cohort studies, cross-sectional studies, observational studies, feasibility studies, pilot studies, and validation studies. Conference abstracts without full-text articles, editorials, opinion papers, narrative reviews, and single-patient case reports were excluded.

The study selection process involved removal of duplicate records followed by screening of titles, abstracts, and full-text articles according to the predefined eligibility criteria. Two independent reviewers (SM and HM) screened each title, abstract, and full-text article to determine study eligibility. Any disagreements were resolved through discussion, and where necessary, a third reviewer (KS) was consulted.

### 2.3 Data extraction

A standardized data extraction form was developed and piloted prior to full data extraction. Two reviewers independently extracted data from the included studies, and any discrepancies were resolved through discussion or consultation with a third reviewer. Extracted data included study characteristics, neurological population, sample size and participant demographics, wearable sensor type and configuration, sensor placement and sampling frequency, digital biomarker characteristics, clinical application, rehabilitation setting, validation methods, measurement properties, primary and secondary outcomes, and key quantitative findings.

To facilitate comparison across studies, an operational glossary of the principal wearable-derived digital biomarkers, including their operational definitions, typical units of measurement, clinical applications, and assessment contexts, is provided in Supplementary Table S2.

### 2.4 Data synthesis

A narrative synthesis approach was used to summarize the findings of the included studies. The findings were organized into thematic categories based on the neurological conditions investigated and the categories of wearable-derived digital biomarkers.

## 3 Results

### 3.1 Literature search

**Figure 1:**
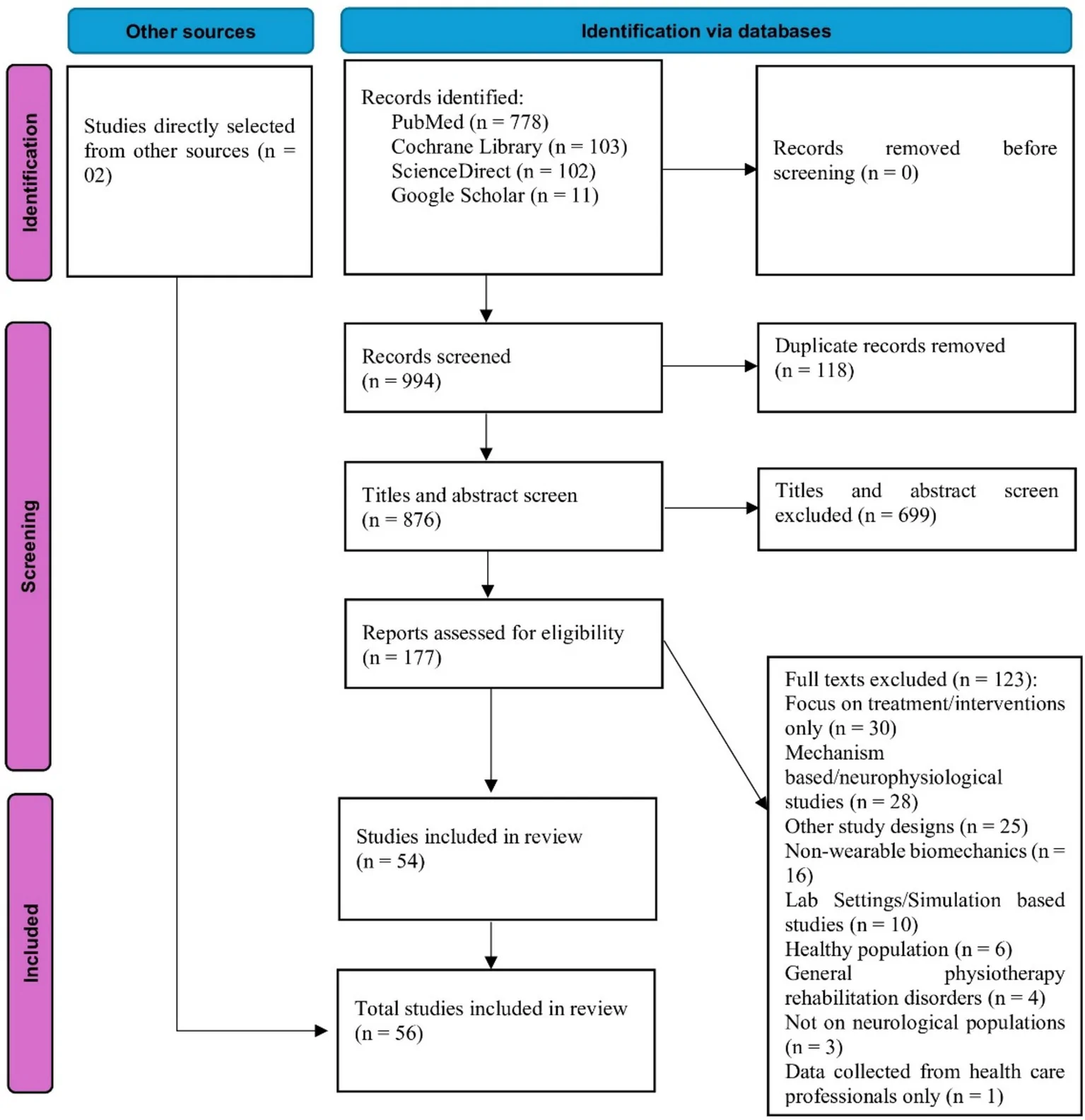
PRISMA flowchart for searching and screening studies.

A systematic search across five databases identified 994 records. After removal of 118 duplicate studies, 876 records remained for title and abstract screening. Following the initial screening, 699 studies were excluded based on the predefined eligibility criteria, resulting in 177 studies undergoing full-text assessment. Of these, 123 full-text articles were excluded for the following reasons: focus on treatment or intervention only (*n* = 30), mechanism-based or neurophysiological studies (*n* = 28), ineligible study designs (*n* = 25), non-wearable biomechanics studies (*n* = 16), laboratory or simulation-based studies (*n* = 10), studies involving healthy populations (*n* = 6), general physiotherapy rehabilitation studies not specific to neurological conditions (*n* = 4), studies not involving neurological populations (*n* = 3), and studies collecting data exclusively from healthcare professionals (*n* = 1). Ultimately, 54 studies met the inclusion criteria and were included in the review, with an additional two studies identified through manual searching, resulting in a total of 56 included studies. The study selection process is illustrated in Figure 1.

### 3.2 Summary of included studies

Among the included studies, a broad range of neurological conditions were represented, including stroke (*n* = 29), Parkinson disease (*n* = 10), spinal cord injury (SCI) (*n* = 5), mixed neurological disorders (*n* = 4), cerebral palsy (*n* = 3), multiple sclerosis (*n* = 3), traumatic brain injury (*n* = 1), and spinocerebellar ataxia (*n* = 1). Validation studies were the most common study design (*n* = 24), followed by cross-sectional studies (*n* = 10) and longitudinal studies (*n* = 9). Most investigations were conducted in European countries, particularly Italy (*n* = 13), and the Netherlands (*n* = 6).

Across all included studies, kinematic biomarkers represented the dominant biomarker category (*n* = 35), particularly in studies investigating gait analysis, balance assessment, postural control, and movement quality. Similarly, inertial measurement units (IMUs) were used in the majority of included studies (*n* = 34), making them the most frequently utilized wearable technology across neurological rehabilitation research. Sensor placement varied according to the neurological condition and intended rehabilitation application, with the trunk, lower back, wrists, ankles, feet, and upper limbs representing the most commonly reported anatomical locations. Sampling frequencies also varied substantially across studies, ranging from 20 Hz to 1,260 Hz, with lower frequencies generally used for daily-life monitoring and higher frequencies applied in laboratory-grade gait and motion analysis systems. The characteristics of the studies included are summarized in Tables 2, 3. A concise summary of the reported psychometric evidence is provided in Supplementary Table S3.

**Table 2:** Methodological characteristics of included studies.

Study	Study design and country	Sample size	Mean age and % female	Sensor type(s)	Number of sensors used	Body placement(s)	Sampling frequency (Hz)	Assessment context
Stroke								
Geerars et al., 2026 (29)	Validation study; Netherlands	105	72.7 ± 12.2; 48.6%	IMU with accelerometer and gyroscope	1	Th8 and L5	104 Hz	Mixed
Wouda et al., 2025 (30)	Cross-sectional study; Netherlands	115	72.25 ± 12.34; 54.8%	IMU	1–3	Upper back, lower back, both feet	104 Hz	Clinical
Felius et al., 2025 (39)	Longitudinal study; Netherlands	35	69.9 ± 10.1; 48.6%	IMU	3	Feet, lower back, ankle	104 Hz (clinical); 52 Hz (daily life)	Mixed
Geerars et al., 2025 (18)	Longitudinal study; Netherlands	94	72 ± 12.2; 57.4%	IMU	1	Upper back (Th8), lower back (L5)	104 Hz	Mixed
Rech et al., 2025 (32)	Cross-sectional study; Brazil	107	NR	IMU	1	Waist (L1-L2)	100 Hz	Laboratory
Salgut et al., 2025 (31)	Cross-sectional study; Turkey	108	64.25 ± 9.52; 40.7%	IMU + force platform	Multiple	Wrist and balance platform	NR	Clinical
Senadheera et al., 2025 (36)	Observational cohort study; Australia	60	61.5 ± 13; 33%	Bilateral wrist accelerometers + smartphone ESM	2	Both wrists	60 Hz	Home/Community
Skvortsov et al., 2025 (75)	Pilot interventional study; Russia	55	59.7 ± 12 yrs.; 20%	IMU	2	Forearm and wrist	NR	Clinical
Somville et al., 2025 (38)	Longitudinal study; Belgium	13	57.1 ± 11.3	3-axis accelerometer + IMU	3	Both wrists and right thigh	30 Hz	Clinical
Gulde et al., 2024 (76)	Cross-sectional study; Germany	50	NR	Smartwatch accelerometer + gyroscope	2	Both wrists	100 Hz accelerometer; 25 Hz gyroscope	Clinical
Cleland et al., 2024 (27)	Validation study; USA	62		IMU	3			Clinical
Tse et al., 2024 (34)	Feasibility pilot study; Australia	12	73 ± 15.6	Accelerometers	2	Both wrists	30 Hz	Clinical
Unger et al., 2024 (77)	Comparative study; Switzerland	15	66.07 ± 14.33; 27%	IMUs + optical motion capture	5	Wrists, upper arms, trunk	120 Hz IMU / 100 Hz OMC	Laboratory
Pregnolato et al., 2023 (78)	Cross-sectional study; Italy	NR	65.09 (12.14); 38%	sEMG + IMU	1	Forearm/hand	NR	Clinical
Schwarz et al., 2023 (25)	Validation study; Switzerland	28	62.04 ± 11.68; 32%	IMU	17	Whole-body anatomical landmarks	1,000 Hz sampling; 60 Hz recording	Clinical
Spina et al., 2023 (33)	Cross-sectional study; Italy	71	NR	IMU	1	Lower back	NR	Clinical
Alhwoaimel et al., 2023 (28)	Validation study; Saudi Arabia	40	–	Mixed	NR	Trunk	NR	Clinical
Daniel et al., 2023 (79)	Validation study; Brazil	24	NR	Triaxial accelerometer	1	Body-worn	NR	Clinical
Hendriks et al., 2022 (26)	Observational cohort study; Netherlands	22	63 ± 13	IMU	2	Ankles	102.4 Hz	Mixed
Werner et al., 2022 (11)	Secondary analysis; Switzerland	21	68 ± 10; 24%	Inertial sensors (accelerometer + gyroscope)	2	Both wrists	50 Hz	Clinical
David et al., 2021 (35)	Observational feasibility study; India	5	35.4 ± 13.2	IMU	2	Both wrists	50 Hz	Home/Community
Meng et al., 2021 (17)	Comparative study; China	23	42 ± 20.94; 43%	Accelerometer, gyroscope, sEMG	6	Upper and lower limb muscles	148 Hz inertial; 1,260 Hz sEMG	Home/Community
Shin et al., 2020 (40)	Longitudinal study; USA	6	NR	IMU + heart-rate monitor	7	Pelvis, thighs, shanks, feet	60 Hz	Clinical
Belluscio et al., 2018 (80)	Validation study; Italy	46	66 ± 16	IMU	5	Distal tibiae, pelvis, sternum, head	NR	Clinical
Held et al., 2018 (10)	Longitudinal study; Netherlands	4	NR	IMU	14	Full body segments	20 Hz	Mixed
Lee et al., 2018 (81)	Validation study; South Korea	24	59.7 ± 14.55; 30%	Accelerometers	4	Bilateral wrists and ankles	30–100 Hz	Laboratory
Lee et al., 2018 (37)	Validation study; USA	30	54.4 ± 10.1	IMU	2	Bilateral wrists	256 Hz	Clinical
Klassen et al., 2017 (82)	Validation study	21	55 ± 10	Activity monitor	2	Nonparetic ankle	NR	Clinical
Bergamini et al., 2017 (9)	Validation study; Italy	70	63 ± 13; 40%	IMU	3	Pelvis, sternum, head	NR	Clinical
Parkinson disease								
De Pasquale et al., 2025 (42)	Observational study; Italy	69	NR	Optoelectronic motion capture + force plates + EMG	Multiple	Lower limbs and trunk	NR	Laboratory
Denk et al., 2022 (13)	Cross-sectional study; Belgium	28	71 (Median age)	IMU + smartphone	2	Shoes and lower back	200 Hz	Mixed
Lencioni et al., 2022 (44)	Validation study; Italy	33	73.9 ± 6.2	Single trunk IMU accelerometer	1	Lower back (L5 trunk)	140 Hz	Laboratory
Lee et al., 2021 (43)	Validation study; Taiwan	20	NR	Inertial sensors	2	Shoes/feet	NR	Home/Community
Albani et al., 2019 (12)	Validation study; Italy	50	66.9; 40%	IMU	3	Bilateral thighs and chest	NR	Home/Community
Botros et al., 2019 (48)	Pilot feasibility study; Switzerland	4	NR	Body-worn + ambient sensors	10	Wrist, hip, ankle	NR	Home/Community
Fiems et al., 2018 (45)	Validation study; USA	30	NR	Smartphone/mobile accelerometer + IMU	2	Mobile device + trunk IMUs	NR	Clinical
Ozinga et al., 2017 (47)	Validation study; USA	54	NR	Mobile inertial sensors	1	Lower back/trunk	NR	Clinical
Bonora et al., 2015 (46)	Validation study; Italy	31	72.5 ± 6.8; 36%	Inertial sensors	2	Trunk (L2-L4) and shank	50 Hz	Laboratory
Mancini et al., 2015 (41)	Observational study; USA	32	65 ± 6	Inertial sensors	3	Lumbar spine (L5) and both feet	NR	Home/Community
Spinal cord injury (SCI)								
Johnson et al., 2025 (52)	Longitudinal study; USA	73	58.6 ± 15.9	IMU	1	Wrist		Clinical
Giagiozis et al., 2025 (15)	RCT; Switzerland, Germany, Spain, Czech Republic	69	49.7 ± 15.1; 15.9%	IMUs	3–5	Wrists, ankles, wheelchair	50 Hz	Home/Community
Marco-Ahulló et al., 2021 (49)	Validation study; Spain	20	45.7 ± 8.37	Accelerometer	1	Non-dominant upper arm	50 Hz	Clinical
Schneider et al., 2018 (50)	Validation study; Switzerland	63	49.3 ± 16.6; 27%	Accelerometers/IMUs	3	Both wrists + wheelchair wheel	50 Hz	Home/Community
Brogioli et al., 2017 (51)	Cross-sectional study; Switzerland	30		Accelerometers	Multiple	Upper limbs / wheelchair monitoring configuration		Mixed
Mixed neurological disorders								
Caronni et al., 2024 (60)	Longitudinal study; Italy	109	78.4 (Median age); 39.5%	IMU	1	Lower trunk	NR	Clinical
Caronni et al., 2023 (61)	Longitudinal study; Italy	214	57.9; 42.1%	IMU	1	Lower trunk	NR	Clinical
Anastasi et al., 2019 (62)	Validation study; Italy	50		IMU	1	Sternum	NR	Clinical
Rauen et al., 2018 (63)	Validation study; Germany	30		Tri-axial accelerometers	Multiple	Trunk and limbs	NR	Clinical
Cerebral palsy								
Kim et al., 2022 (56)	Validation study; South Korea	21	11.10 ± 6.28	Wireless postural sway sensor	1	Trunk/body posture placement	NR	Clinical
Gerber et al., 2021 (55)	Validation study; Switzerland	29	13.5 ± 3.4; 60%	IMU	5	Thighs, shanks, trunk	100 Hz	Mixed
Carcreff et al., 2019 (57)	Cross-sectional study; Switzerland	20		Inertial sensors	4	Bilateral shanks and thighs	100 Hz	Mixed
Multiple sclerosis								
Block et al., 2022 (14)	Longitudinal study; USA	94	50.0 ± 13.6; 62% years	Wrist-worn accelerometer	1	Non-dominant wrist	NR	Mixed
Tanoh et al., 2021 (54)	Validation study; France	116		Smartphone embedded sensors	1	Hand-held smartphone	NR	Clinical
Flachenecker et al., 2020 (53)	Validation study; Germany	124		Foot-worn inertial sensors	2	Feet	NR	Clinical
Traumatic brain injury								
Arippa et al., 2024 (58)	Cross-sectional study; Italy	36	37.4 ± 17.4	Wearable inertial sensor + pressure platform	1	Waist (L4-L5)	NR	Clinical
Spinocerebellar ataxia								
Velázquez-Pérez et al., 2020 (59)	Case–control study; Cuba	30	NR	Wearable inertial sensors	Multiple	Lumbar trunk, sternum, arms	NR	Laboratory

NR, Not reported.

**Table 3:** Characteristics and clinical interpretation of wearable-derived biomarkers across neurological disorders.

Study	Primary clinical application	Biomarker	Domain	Unit	Validity findings	Clinical interpretation
Stroke						
Geerars et al., 2026 (29)	Balance assessment	Sway score	Kinematic	*z*-score	Reliable with low floor/ceiling effects	Complementary
Wouda et al., 2025 (30)	Prediction of Activities of Daily Living independence and walking ability	Postural sway path	Kinematic	mm/s^4^	Good–excellent reliability	Mixed sensitivity
Felius et al., 2025 (39)	Prediction of community walking after stroke	Strides per day	Activity-based	strides/day	Adjusted R^2^ = 0.60	Complementary
Geerars et al., 2025 (18)	Postural sway monitoring	Postural sway path	Kinematic	mm/s	Limited responsiveness	Limited sensitivity
Rech et al., 2025 (32)	Mobility smoothness and safety monitoring	Spectral arc length	Kinematic	Smoothness metric	Significant group differences	Complementary
Salgut et al., 2025 (31)	Balance and fall-risk assessment	Center of pressure displacement	Kinetic	mm	Correlated with BBS	Complementary
Senadheera et al., 2025 (36)	Upper-limb activity participation monitoring	Affected arm activity ratio	Activity-based	ratio	NR	Complementary
Skvortsov et al., 2025 (75)	Wrist-function assessment	Wrist motion amplitude	Kinematic	degrees	4–10° improvement after FES	Complementary
Somville et al., 2025 (38)	Motor activity monitoring	Magnitude ratio	Activity-based	ratio	High correlation with dexterity	Complementary
Gulde et al., 2024 (76)	Upper-limb laterality and activity monitoring	Walking speed	Kinematic	m/s	ICC ≥ 0.94	Complementary
Cleland et al., 2024 (27)	Walking speed assessment	Mean amplitude deviation	Activity-based	mg	Associated with impairment	Complementary
Tse et al., 2024 (34)	Upper-limb activity monitoring	Upper-limb activity use	Activity-based	hours/day	rs = 0.71	Complementary
Unger et al., 2024 (77)	Upper-limb movement quality assessment	End-effector velocity	Kinematic	mm/s	Strong IMU-OMC agreement	Complementary
Pregnolato et al., 2023 (78)	Hand motor rehabilitation training	Hand gesture control	Kinematic	Number of gestures	NR	Complementary
Schwarz et al., 2023 (25)	Gait analysis and rehabilitation assessment	Walking speed	Kinematic	m/s	ICC up to 0.90	Complementary
Spina et al., 2023 (33)	Turning mobility quantification	Average turning speed	Kinematic	degrees/s	AUC 0.742–0.912	Complementary
Alhwoaimel et al., 2023 (28)	Objective assessment of trunk movement during upper limb tasks	Trunk range of motion (ROM)	Kinematic	Degrees / ROM measures	ICC = 0.71–0.92	Complementary
Daniel et al., 2023 (79)	Energy expenditure estimation	Energy expenditure	Physiological	NR	ICC = 0.94	Complementary
Hendriks et al., 2022 (26)	Gait capacity and gait performance monitoring	Capacity-based gait speed	Kinematic	m/s	r ≥ 0.81	Complementary
Werner et al., 2022 (11)	Upper-limb movement quality estimation	ARAT total score	Kinematic	0–57 score	MAE = 2.9; r^2^ = 0.93	Complementary
David et al., 2021 (35)	Real-world arm-use quantification	Gross Movement Score	Activity-based	Binary score	Accuracy 50–60%	Limited sensitivity
Meng et al., 2021 (17)	Human activity recognition	Human activity recognition (HAR) classification accuracy	Activity-based	Percent accuracy	95.84 ± 1.75%	Complementary
Shin et al., 2020 (40)	Quantification of physical therapy dosage	Amount of motion (AoM)	Kinematic	Degrees	r^2^ = 32.1%	Complementary
Belluscio et al., 2018 (80)	Dynamic balance assessment	RMS acceleration	Kinematic	Acceleration metrics	Non-significant group differences	Complementary
Held et al., 2018 (10)	Daily-life upper-limb kinematics	Reaching counts	Activity-based	counts	Responsive to recovery changes	Complementary
Lee et al., 2018 (81)	Physical activity monitoring during rehabilitation	Physical activity energy expenditure	Physiological	kcal/min	ICC = 0.953–0.980	Complementary
Lee et al., 2018 (37)	Home/community upper-limb rehabilitation	Goal-directed movement count	Activity-based	Classification probability	C-statistic 87.0%	Complementary
Klassen et al., 2017 (82)	Walking intensity and step-count monitoring	Step count	Activity-based	Steps	Slope 0.99 vs. SAM	Complementary
Bergamini et al., 2017 (9)	Gait stability and fall risk assessment	Acceleration attenuation	Kinematic	Acceleration indices	NR	Complementary
Parkinson disease						
De Pasquale et al., 2025 (42)	Biomechanical gait dysfunction assessment	Stride length	Kinematic	m	Statistical difference (*p* < 0.001)	Complementary
Denk et al., 2022 (13)	Freezing of gait quantification	Percentage Time Frozen	Kinematic	%TF	Moderate correlations with provoking tests	Complementary
Lencioni et al., 2022 (44)	Anticipatory postural adjustment detection	APA onset	Kinematic	Seconds	R = 0.99 vs. force platform	Complementary
Lee et al., 2021 (43)	Gait assessment reliability	Stride parameters	Kinematic	Spatiotemporal gait metrics	ICC 0.64–0.87	Complementary
Albani et al., 2019 (12)	Remote monitoring and automated UPDRS assessment	Gait	Kinematic	NR	ICC = 0.61	Complementary
Botros et al., 2019 (48)	Long-term home monitoring	Daily wear time	Activity-based	Hours/day	15 h/day average	Complementary
Fiems et al., 2018 (45)	Measurement of postural sway	Sway Balance score	Kinematic	Balance score	ICC = 0.72–0.92	Complementary
Ozinga et al., 2017 (47)	Postural stability assessment	Peak sway acceleration	Kinematic	m/s^2^	NR	Complementary
Bonora et al., 2015 (46)	Assessment of anticipatory postural adjustments	ML trunk acceleration	Kinematic	Acceleration	NR	Complementary
Mancini et al., 2015 (41)	Turning mobility monitoring	Turn mean velocity	Kinematic	Degrees/s	Sensitive to PD-related impairment: 43.3 ± 4.8°/s vs. 38 ± 5.7°/s	Complementary
Spinal cord injury (SCI)						
Johnson et al., 2025 (52)	Upper extremity function monitoring	Distance correlation features	Kinematic	dCorr	dCorr 0.50–0.72	Complementary
Giagiozis et al., 2025 (15)	Continuous physical activity monitoring	Average daily energy expenditure	Physiological	kcal/day	Strong correlations with SCIM (*r* = 0.69) and UEMS (*r* = 0.63)	Complementary
Marco-Ahulló et al., 2021 (49)	Energy expenditure estimation	Energy expenditure	Physiological	Oxygen consumption/EE	*r* = 0.72	Complementary
Schneider et al., 2018 (50)	Physical activity reliability monitoring	Activity counts	Activity-based	Counts/min	High reliability	Complementary
Brogioli et al., 2017 (51)	Assessment of upper-limb activity and independence during rehabilitation	Upper-extremity activity count	Activity-based	Activity counts	Correlated with neurological level and independence	Complementary
Mixed neurological disorders						
Caronni et al., 2024 (60)	Dynamic balance assessment	Gait speed	Kinematic	m/s	MDC = 0.13 m/s	Complementary
Caronni et al., 2023 (61)	Inpatient rehabilitation, Italy	Turning duration	Kinematic	Seconds	AUC = 0.69	Complementary
Anastasi et al., 2019 (62)	Position change monitoring	Gait	Kinematic	PCA-derived score	*r* = 0.84	Complementary
Rauen et al., 2018 (63)	Balance and fall-risk assessment	Position change detection	Activity-based	% detected	100% detection	Complementary
Cerebral palsy						
Kim et al., 2022 (56)	Dynamic postural sway and fall-risk assessment	Dynamic postural sway	Kinematic	Sway metric	*k* = 0.973	Complementary
Gerber et al., 2021 (55)	Daily-life gait and activity monitoring	Stride velocity	Kinematic	m/s	ICC up to 0.98	Complementary
Carcreff et al., 2019 (57)	Walking bout detection	Walking bouts	Activity-based	Bout counts	Higher sensitivity/accuracy	Complementary
Multiple sclerosis						
Block et al., 2022 (14)	Remote fall-risk monitoring	Daily step count	Activity-based	Steps/day	Associated with falls (*p* = 0.007)	Complementary
Tanoh et al., 2021 (54)	Self-assessment digital biomarkers	MSCopilot Walking score	Kinematic	Digital score	*r* = 0.65 with EDSS	Complementary
Flachenecker et al., 2020 (53)	Objective gait impairment assessment	Stride length	Kinematic	cm	*r* = 0.955	Complementary
Traumatic brain injury						
Arippa et al., 2024 (58)	Functional mobility and postural control assessment	Sway area	Kinematics	mm^2^	*p* = 0.003	Complementary
Spinocerebellar ataxia						
Velázquez-Pérez et al., 2020 (59)	Gait and postural sway biomarker assessment	Swing time variability	Kinematic	% variability	Correlated with onset	Complementary

NR, Not reported.

### 3.3 Wearable-derived biomarkers in stroke rehabilitation

**Figure 2:**
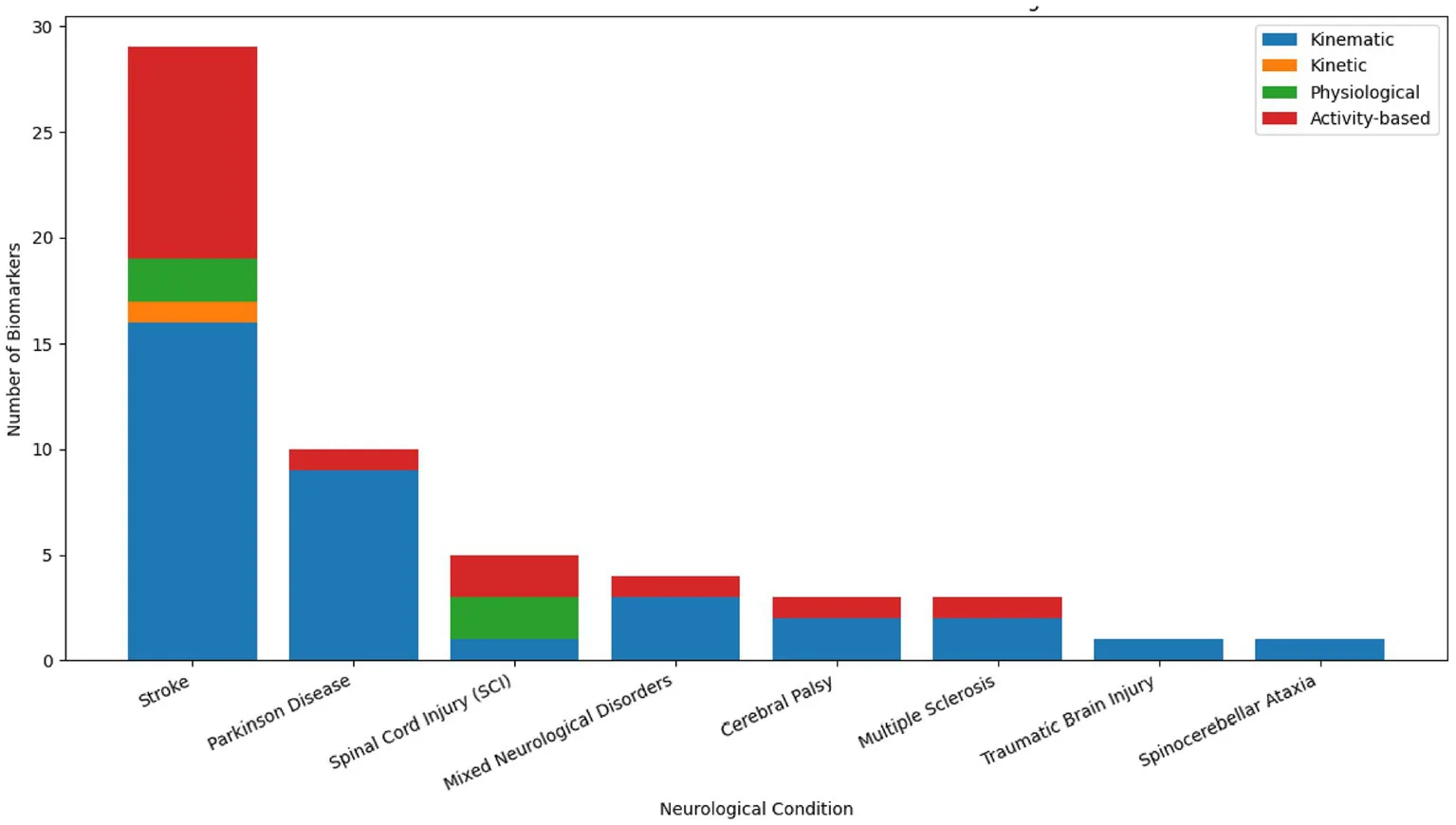
Distribution of wearable-derived primary biomarker domains across neurological conditions.

Stroke was the most frequently investigated neurological condition (*n* = 29) with 20 studies which utilize IMU-based or inertial sensor systems, either as standalone devices or in combination with other wearable sensors. Furthermore, kinematic biomarkers were the predominant biomarker domain in stroke rehabilitation (16/29) (Figure 2).

For wearable-derived gait and balance metrics, several studies demonstrated strong validity and reliability. Schwarz et al. (2023) reported excellent reliability (ICC = 0.90) for wearable-based gait analysis (25), while Hendriks et al. (2022) demonstrated strong correlations between wearable-derived gait speed and clinical gait performance measures (*r* ≥ 0.81) (26). Similarly, Cleland et al. (2024) demonstrated excellent agreement between wearable-derived and stopwatch-based walking speed measurements, with ICC values ≥ 0.94 (27). Alhwoaimel et al. (2023) also reported favorable validity findings, including ICC values ranging from 0.71 to 0.92 for trunk range-of-motion assessment (28).

Balance and postural control also emerged as major research focuses within stroke rehabilitation studies. Geerars et al. (2026) demonstrated that sway-score biomarkers provided reliable balance assessment with low floor and ceiling effects (29), whereas Geerars et al. (2025) later reported limited responsiveness of postural sway measures during rehabilitation (18). Wouda et al. (2025) identified good-to-excellent reliability of postural sway metrics for predicting activities of daily living independence and walking ability after stroke (30). Similarly, Salgut et al. (2025) reported significant correlations between center-of-pressure displacement and Berg Balance Scale scores, supporting the use of wearable-derived balance biomarkers for fall-risk assessment (31).

There were studies which also focused on mobility quality and movement smoothness during functional activities. Rech et al. (2025) demonstrated significant differences (*p* < 0.05) in spectral arc length metrics between stroke populations and controls, indicating the potential utility of smoothness-based biomarkers for mobility safety monitoring (32). Spina et al. (2023) also demonstrated strong discriminative performance of turning-speed biomarkers, with AUC values ranging from 0.742 to 0.912 during instrumented mobility assessment (33).

Upper-limb rehabilitation and real-world arm-use monitoring emerged as another major application area. Accelerometer- and IMU-based systems were used to quantify upper-limb activity, movement quality, rehabilitation participation, and community-based arm use (34–36). Frequently reported activity-based biomarkers included affected arm activity ratio, reaching counts, upper-limb activity use, goal-directed movement counts, and magnitude ratios (10, 37, 38). These studies also demonstrated promising associations between wearable-derived activity measures and functional recovery outcomes. For example, Somville et al. (2025) reported strong correlations between accelerometer-derived magnitude ratios and dexterity-related recovery (38), while Lee et al. (2018) demonstrated strong classification performance for goal-directed movement monitoring (C-statistic = 87.0%) (37).

A small number of studies also explored wearable-derived biomarkers for rehabilitation monitoring and prediction of functional recovery. Felius et al. (2025) demonstrated that stride counts were associated with community walking outcomes after stroke (adjusted *R*^2^ = 0.60) (39), while Shin et al. (2020) used wearable lower-body kinematics to longitudinally quantify physical therapy dosage during early rehabilitation (r^2^ = 32.1%) (40).

### 3.4 Wearable-derived biomarkers in Parkinson disease

Parkinson disease (PD) was the second most commonly investigated neurological condition in 10/56 of included studies. Similar to stroke-related research, IMU-based or inertial sensor systems were the predominant wearable technologies in four studies and inertial sensor systems reported in three additional studies. Moreover, kinematic biomarkers were the dominant biomarker category in PD research, accounting for 9 of the 10 identified biomarkers (Figure 2).

Gait dysfunction and mobility impairment were the principal research themes across PD studies. For instance, Denk et al. (2022) demonstrated moderate correlations between wearable-derived percentage time frozen and conventional provocation tests, supporting the utility of wearable systems for detecting freezing episodes during daily-life activities (13). Similarly, Mancini et al. (2015) reported that wearable-derived turn mean velocity was sensitive to PD-related mobility impairment, with slower turning velocities observed in individuals with Parkinson disease (43.3 ± 4.8°/s versus 38 ± 5.7°/s) (41). De Pasquale et al. (2025) additionally identified significant differences in stride-length biomarkers during biomechanical gait assessment (*p* < 0.001) (42).

Apart from gait dysfunction and mobility impairment, several PD investigations demonstrated favorable validity and reliability of wearable-derived gait and balance measures. Lee et al. (2021) reported moderate-to-good reliability for foot-worn gait parameters, with ICC values ranging from 0.64 to 0.87 (43). Similarly, Lencioni et al. (2022) demonstrated near-perfect agreement between wearable-derived anticipatory postural adjustment (APA) detection and force-platform assessment (*R* = 0.99) (44). Likewise, Fiems et al. (2018) demonstrated acceptable validity of wearable-derived sway balance scores, with ICC values ranging from 0.72 to 0.92 (45). In addition, Bonora et al. (2015) investigated wearable-derived biomarkers related to anticipatory postural adjustments and gait initiation (46), whereas Ozinga et al. (2017) evaluated wearable-derived sway and postural stability measures (47).

Unlike several other neurological populations, PD studies more frequently incorporated long-term home monitoring and adherence-related outcomes. Botros et al. (2019) demonstrated successful long-term home monitoring using body-worn and ambient sensor systems, with participants achieving an average daily wear time of approximately 15 h/day (48). Similarly, Albani et al. (2019) explored remote multi-sensor monitoring approaches for automated gait and UPDRS-related assessment in PD populations, reporting moderate agreement (ICC = 0.61) (12).

### 3.5 Wearable-derived biomarkers in spinal cord injury

Unlike stroke and PD studies, SCI-related investigations (*n* = 5) primarily focused on activity-based (*n* = 2) and physiological biomarkers (*n* = 2), whereas only one study investigated a predominantly kinematic biomarker. These biomarkers were mainly explored for physical activity monitoring, rehabilitation participation, independence assessment, and upper-extremity functional evaluation. Moreover, accelerometer-based monitoring was the most commonly utilized sensing approach across these studies.

Two studies specifically investigated wearable-derived energy expenditure measures in SCI populations. Giagiozis et al. (2025) demonstrated strong correlations between daily energy expenditure and established clinical measures, including the Spinal Cord Independence Measure (SCIM) (*r* = 0.69) and Upper Extremity Motor Score (UEMS) (*r* = 0.63) (15). Similarly, Marco-Ahulló et al. (2021) reported significant associations between wearable-derived energy expenditure estimates and oxygen-consumption measures (*r* = 0.72) (49).

Activity monitoring also emerged as a major research focus within SCI studies. Schneider et al. (2018) demonstrated high reliability of wearable-derived activity counts during wheelchair-based physical activity monitoring (50). Brogioli et al. (2017) reported significant associations between upper-extremity activity counts, neurological level, and functional independence (51). Lastly, Johnson et al. (2025) investigated the upper-extremity function monitoring through wearable-derived distance-correlation features and also reported moderate-to-strong associations (dCorr = 0.50–0.72) (52).

### 3.6 Wearable-derived biomarkers in other neurological conditions

Compared with stroke and PD research, other neurological populations were considerably underrepresented; however, several studies reported promising findings.

In multiple sclerosis studies, kinematic biomarkers were the predominant biomarker category (*n* = 2/3), with wearable technologies mainly applied for gait impairment assessment and remote disability monitoring. Flachenecker et al. (2020) demonstrated strong correlations between wearable-derived stride-length metrics and clinical gait impairment (*r* = 0.955) (53). Similarly, Tanoh et al. (2021) reported moderate associations between smartphone-derived MSCopilot walking scores and disability severity measured using the Expanded Disability Status Scale (EDSS) (*r* = 0.65) (54). Block et al. (2022) additionally identified significant associations between daily step counts and fall risk (*p* = 0.007), suggesting the potential utility of activity-based biomarkers for remote monitoring in MS populations (14).

In cerebral palsy (CP) studies, wearable-derived biomarkers were mainly investigated for gait monitoring, walking-bout detection, and dynamic postural sway assessment. Kinematic biomarkers accounted for two of the three identified biomarkers within CP studies. Gerber et al. (2021) demonstrated excellent reliability of wearable-derived stride-velocity monitoring in children with cerebral palsy, reporting ICC values up to 0.98 (55). Kim et al. (2022) demonstrated excellent agreement for dynamic postural sway assessment (*κ* = 0.973) (56). In addition, Carcreff et al. (2019) reported improved sensitivity and accuracy of wearable-based walking-bout detection during real-world monitoring (57).

Evidence related to traumatic brain injury (TBI) and spinocerebellar ataxia remained limited. Arippa et al. (2024) investigated wearable-derived sway-area biomarkers for functional mobility and postural control assessment following severe TBI and identified significant differences during balance evaluation (*p* = 0.003) (58). Likewise, Velázquez-Pérez et al. (2020) demonstrated that wearable-derived swing-time variability was associated with disease onset and gait impairment in individuals with spinocerebellar ataxia (59).

Studies involving mixed neurological disorders (*n* = 4) also predominantly investigated kinematic biomarkers (*n* = 3/4). Caronni et al. (2024) demonstrated clinically meaningful measurement properties for wearable-derived gait-speed assessment, reporting a minimal detectable change (MDC) value of 0.13 m/s (60). Similarly, Caronni et al. (2023) demonstrated acceptable discriminative performance of turning-duration biomarkers for fall-risk assessment (AUC = 0.69) (61). Anastasi et al. (2019) reported strong correlations between wearable-derived gait biomarkers and clinical performance measures (*r* = 0.84) (62), while Rauen et al. (2018) demonstrated 100% accuracy for wearable-based position-change detection in immobile neurological patients (63).

## 4 Discussion

The findings of this scoping review (*n* = 56) demonstrate that wearable-derived digital biomarkers are increasingly being used to complement conventional rehabilitation assessments through continuous, objective, and ecologically valid measurement of physical function. However, due to substantial heterogeneity across the included studies, the clinical implementation of wearable-derived biomarkers in neurological rehabilitation remains in the early stages of development.

Stroke accounted for the largest proportion of studies (*n* = 29) included in this review which reflects both the global burden of stroke-related disability (64) and the increasing clinical need for objective monitoring throughout the recovery process (5, 65). Moreover, the dominance of kinematic biomarkers in stroke-related studies reflects the current emphasis on gait assessment and movement quality within neurorehabilitation (66, 67). These findings are clinically important because gait impairment and postural instability are major factors contributing to long-term disability, increased fall risk, and reduced participation after stroke (68).

Parkinson disease studies demonstrated a different clinical emphasis compared with stroke rehabilitation research. While stroke studies largely focused on recovery monitoring and rehabilitation participation, PD studies primarily investigated disease-specific motor impairments. Another important finding in case of PD was the increasing use of long-term home monitoring and ecological assessment in PD populations. In contrast to many stroke studies that were primarily conducted in clinical settings, several PD investigations implemented wearable systems for prolonged home-based monitoring and continuous mobility tracking (12, 48). This likely reflects the fluctuating nature of Parkinson symptoms, including variations in gait impairment and freezing episodes across different environments and times of day (69).

Compared with stroke and PD, wearable biomarker applications in SCI demonstrated notably different rehabilitation priorities. Activity-based and physiological biomarkers were the predominant domains in SCI studies, contrasting with the kinematic biomarker dominance observed in stroke and PD populations. These findings are aligned with the distinct rehabilitation goals in SCI, where maintaining upper-limb function, promoting physical activity participation, enhancing independence, and supporting wheelchair mobility are often prioritized (70, 71). However, most SCI investigations were observational or validation-based, with only five studies identified, thereby limiting understanding of the long-term clinical utility and implementation of wearable-derived biomarkers. Similar to SCI, studies involving other neurological diseases were also underrepresented within the literature, highlighting the need for further high-quality research across diverse neurological populations.

In terms of sensor technologies, 34 studies utilized IMU-based systems either as standalone devices or in combination with other wearable sensors. The widespread use of IMU-based technologies reflects their portability, relatively low cost, and ability to capture multidimensional movement data across both clinical and real-world environments (72, 73). Unlike conventional laboratory-based motion analysis systems, IMUs enable continuous monitoring of gait, balance, mobility, and upper-limb function without requiring specialized infrastructure (73, 74). In addition, their portability facilitates home- and community-based assessment, supporting ecological monitoring of functional performance during daily-life activities (74).

The findings of this review have several important clinical implications for neurological rehabilitation practice. Wearable technologies may support remote monitoring and telerehabilitation, particularly for individuals requiring long-term follow-up or community-based rehabilitation. In addition, wearable-derived biomarkers may facilitate more personalized rehabilitation approaches through continuous assessment of recovery trajectories, rehabilitation dosage, mobility performance, and participation patterns.

Despite these potential advantages, several methodological limitations and evidence gaps within the current literature should be acknowledged. Considerable heterogeneity was identified across the included studies regarding sensor placement, acquisition frequencies, algorithm development, biomarker definitions, and validation methods. Moreover, the majority of included studies were small-sample observational or validation-based investigations, resulting in limited evidence regarding responsiveness to rehabilitation-related changes. There were only 9 of the 56 included studies (16.1%) which employed a longitudinal design to evaluate changes over time. In addition, evidence regarding user acceptance and usability was also limited, as only a small number of the included studies evaluated outcomes such as usability, adherence, or user experience. Finally, several neurological populations remained underrepresented within the literature, particularly traumatic brain injury, spinocerebellar ataxia, and pediatric neurorehabilitation populations.

Based on these limitations, future research should prioritize the standardization of wearable-derived biomarker definitions, sensor protocols, and validation methodologies across neurological rehabilitation populations. Additional large longitudinal and multicenter studies are needed to further establish the clinical utility of wearable-derived biomarkers for predicting recovery and supporting individualized intervention planning. Future research should also incorporate user-centered outcomes alongside psychometric validation to support the successful implementation of wearable-derived digital biomarkers in neurological rehabilitation. Furthermore, future investigations should focus on multimodal monitoring approaches that combine kinematic, physiological, and activity-based biomarkers within real-world rehabilitation environments.

## 5 Conclusion

There is increasing integration of wearable technologies, particularly IMU-based systems, for objective evaluation of gait, balance, mobility, upper-limb function, and rehabilitation participation across both clinical and real-world settings. Kinematic biomarkers were the most frequently investigated domain within the included studies, especially in stroke and Parkinson disease populations, whereas activity-based and physiological biomarkers were more prominent in spinal cord injury research.

Overall, the available evidence indicates that wearable-derived digital biomarkers have significant potential to complement traditional rehabilitation assessments by enabling continuous, objective, and ecologically valid monitoring of physical function. The expanding use of wearable systems in home- and community-based settings further emphasizes their potential applications in remote monitoring, telerehabilitation, and personalized rehabilitation planning. Nevertheless, despite these promising findings, substantial heterogeneity across the included studies suggests that the clinical implementation of wearable-derived biomarkers in neurological rehabilitation remains at an early stage of development.
