# Supplementary material for: Digital biomarkers derived from wearable sensors in neurorehabilitation: a scoping review
Source: Front Neurol. 2026 Sep 3;17:1908796. doi: 10.3389/fneur.2026.1908796 (PMC13581664; doi:10.3389/fneur.2026.1908796)
Supplement: Supplementary file 1 [file Supplementary_file_1.docx]

Table 1. Search Strategy

| Database | Search Strings | No. of Results |
| --- | --- | --- |
|  | (("digital biomarker*"[All Fields] OR "digital biometrics"[All Fields] OR "wearable sensor*"[All Fields] OR "wearable device*"[All Fields] OR "inertial sensor*"[All Fields] OR "inertial measurement unit*"[All Fields] OR "IMU"[All Fields] OR "accelerometer*"[All Fields] OR "gyroscope*"[All Fields] OR "electromyograph*"[All Fields] OR "smart insole*"[All Fields] OR "body worn sensor*"[All Fields]) AND ("neurorehabilitation"[All Fields] OR "neurological rehabilitation"[All Fields] OR "motor rehabilitation"[All Fields] OR "physical rehabilitation"[All Fields] OR "functional recovery"[All Fields]) AND ("stroke"[All Fields] OR "parkinson*"[All Fields] OR "multiple sclerosis"[All Fields] OR "traumatic brain injury"[All Fields] OR "TBI"[All Fields] OR "spinal cord injury"[All Fields] OR "SCI"[All Fields] OR "cerebral palsy"[All Fields])) AND ((2015/1/1:2026/5/4[pdat]) AND (english[Filter])) | 778 |
| ScienceDirect | TITLE-ABS-KEY ("digital biomarker" OR "digital biometrics" OR "wearable sensor" OR "wearable device" OR "inertial sensor" OR "inertial measurement unit") AND ("motor rehabilitation" OR "physical rehabilitation" OR "functional recovery") 2015-2026 | 41 |
|  | TITLE-ABS-KEY ("digital biomarker" OR "digital biometrics" OR "wearable sensor" OR "wearable device" OR "inertial sensor" OR "inertial measurement unit") AND ("motor rehabilitation" OR "physical rehabilitation" OR "functional recovery") 2015-2026 | 26 |
|  | TITLE-ABS-KEY ("IMU" OR "accelerometer"OR "gyroscope" OR "electromyograph" OR "smart insole" OR "body-worn sensor) AND ("neurorehabilitation" OR "neurological rehabilitation") 2015-2026 | 13 |
|  | TITLE-ABS-KEY ("IMU" OR "accelerometer"OR "gyroscope" OR "electromyograph" OR "smart insole" OR "body-worn sensor) AND ("motor rehabilitation" OR "physical rehabilitation" OR "functional recovery") 2015-2026 | 22 |
| Cochrane Library | ("digital biomarker" OR "digital biometrics" OR "wearable sensor" OR "wearable device" OR "inertial sensor" OR "inertial measurement unit" OR "IMU" OR "accelerometer" OR "gyroscope" OR "electromyograph*" OR "smart insole" OR "body-worn sensor") AND ("neurorehabilitation" OR "neurological rehabilitation" OR "motor rehabilitation" OR "physical rehabilitation" OR "functional recovery") AND ("stroke" OR "parkinson" OR "multiple sclerosis" OR "traumatic brain injury" OR "TBI" OR "spinal cord injury" OR "SCI" OR "cerebral palsy") in Title Abstract Keyword | 103 |
| Google Scholar | allintitle: ("digital biomarker*" OR "digital biometrics" OR "wearable sensor*" OR "wearable device*" OR "inertial sensor*" OR "inertial measurement unit*" OR "IMU" OR "accelerometer*" OR "gyroscope*" OR "electromyograph*" OR "smart insole*" OR "body-worn sensor*") AND ("neurorehabilitation" OR "neurological rehabilitation" OR "motor rehabilitation" OR "physical rehabilitation" OR "functional recovery") | 11 |

Table 2. Operational glossary of the principal wearable-derived digital biomarkers identified in the included studies

| Biomarker | Operational Definition | Typical Calculation Method / Algorithm | Typical Unit | Typical Sensor Placement | Typical Sampling Frequency | Primary Clinical Application | Typical Assessment Context |
| --- | --- | --- | --- | --- | --- | --- | --- |
| Gait speed | Average walking speed during ambulation. | Distance travelled ÷ walking time. | m/s | Lower back, feet, shanks | 50–100 Hz | Gait assessment | Laboratory and home/community |
| Stride length | Distance covered in one stride. | Estimated from IMU gait-event detection algorithms. | m | Feet, shanks, lower back | 50–100 Hz | Gait assessment | Laboratory and home/community |
| Cadence | Number of steps taken per minute. | Step detection algorithm (steps/min). | Steps/min | Feet, shanks, lower back | 50–100 Hz | Walking performance | Laboratory and home/community |
| Step count | Total number of steps recorded during monitoring. | Step detection algorithm. | Steps/day | Wrist, ankle, waist | 20–100 Hz | Physical activity monitoring | Home/community |
| Turning speed | Angular velocity during turning movements. | Derived from gyroscope angular velocity signals. | °/s | Lower back/trunk | 100 Hz | Mobility and fall-risk assessment | Laboratory |
| Turning duration | Time required to complete a turn. | Time elapsed between turn onset and completion. | s | Lower back/trunk | 100 Hz | Functional mobility | Laboratory |
| Postural sway | Magnitude of body sway during standing balance. | Acceleration or angular displacement analysis. | mm, sway score | Lower back/trunk | 50–100 Hz | Balance assessment | Laboratory and clinical |
| Centre of pressure (COP) displacement | Displacement of the body's centre of pressure. | Estimated from force platform or validated IMU algorithms. | mm | Lower back/trunk | 100 Hz | Balance assessment | Laboratory |
| Movement smoothness | Quality of movement during functional tasks. | Spectral Arc Length (SPARC) or jerk-based algorithms. | Unitless | Wrist, upper limb | 50–100 Hz | Upper-limb assessment | Laboratory |
| Range of motion (ROM) | Angular excursion of a joint during movement. | Joint angle estimation from IMU orientation. | Degrees (°) | Upper limb or trunk | 50–100 Hz | Upper-limb assessment | Laboratory |
| Affected arm activity ratio | Relative use of the affected compared with the unaffected arm. | Ratio of movement counts or activity duration between limbs. | Ratio | Bilateral wrists | 30–100 Hz | Stroke rehabilitation | Home/community |
| Magnitude ratio | Relative movement intensity between upper limbs. | Log-transformed ratio of acceleration magnitude. | Ratio | Bilateral wrists | 30–100 Hz | Upper-limb activity monitoring | Home/community |
| Energy expenditure | Estimated energy expenditure during activity. | Accelerometer-based prediction equations or regression models. | kcal/day or METs | Wrist, ankle, waist | 30–100 Hz | Physical activity monitoring | Home/community |
| Daily wear time | Total duration the wearable device is worn each day. | Time recorded by device logs. | Hours/day | Device-dependent | Device-dependent | Feasibility/adherence | Home/community |

COP, centre of pressure; IMU, inertial measurement unit; METs, metabolic equivalents; SPARC, Spectral Arc Length.

Note: The calculation methods, sensor placements, and sampling frequencies shown are typical approaches identified across the included studies and are intended to provide a general operational overview. Study-specific details are reported in Table 2 of the main manuscript.

Table 3. Summary of the reported psychometric evidence for wearable-derived digital biomarkers across the included studies

| Study | Neurological Condition | Biomarker | Reliability | Validity | Responsiveness |
| --- | --- | --- | --- | --- | --- |
| Stroke |  |  |  |  |  |
| Geerars et al., 2026 | Stroke | Sway score | Reliable | Low floor/ceiling effects | NR |
| Wouda et al., 2025 | Stroke | Postural sway path | Good–excellent reliability | Mixed sensitivity | NR |
| Felius et al., 2025 | Stroke | Strides/day | NR | Adjusted R² = 0.60 | NR |
| Geerars et al., 2025 | Stroke | Postural sway path | NR | NR | Limited responsiveness |
| Rech et al., 2025 | Stroke | Spectral arc length | NR | Significant group differences | NR |
| Salgut et al., 2025 | Stroke | Center of pressure displacement | NR | Correlated with BBS | NR |
| Senadheera et al., 2025 | Stroke | Affected arm activity ratio | NR | NR | NR |
| Skvortsov et al., 2025 | Stroke | Wrist motion amplitude | NR | 4–10° improvement after FES | NR |
| Somville et al., 2025 | Stroke | Magnitude ratio | NR | High correlation with dexterity | NR |
| Gulde et al., 2024 | Stroke | Walking speed | ICC ≥ 0.94 | NR | NR |
| Cleland et al., 2024 | Stroke | Mean amplitude deviation | NR | Associated with impairment | NR |
| Tse et al., 2024 | Stroke | Upper-limb activity use | NR | rs = 0.71 | NR |
| Unger et al., 2024 | Stroke | End-effector velocity | NR | Strong IMU–OMC agreement | NR |
| Pregnolato et al., 2023 | Stroke | Hand gesture control | NR | NR | NR |
| Schwarz et al., 2023 | Stroke | Walking speed | ICC up to 0.90 | NR | NR |
| Spina et al., 2023 | Stroke | Average turning speed | NR | AUC 0.742–0.912 | NR |
| Alhwoaimel et al., 2023 | Stroke | Trunk ROM | ICC = 0.71–0.92 | NR | NR |
| Daniel et al., 2023 | Stroke | Energy expenditure | ICC = 0.94 | NR | NR |
| Hendriks et al., 2022 | Stroke | Capacity-based gait speed | NR | r ≥ 0.81 | NR |
| Werner et al., 2022 | Stroke | ARAT total score | NR | MAE = 2.9; r² = 0.93 | NR |
| David et al., 2021 | Stroke | Gross Movement Score | NR | Accuracy 50–60% | NR |
| Meng et al., 2021 | Stroke | HAR classification accuracy | NR | Accuracy 95.84 ± 1.75% | NR |
| Shin et al., 2020 | Stroke | Amount of motion | NR | r² = 32.1% | NR |
| Belluscio et al., 2018 | Stroke | RMS acceleration | NR | Non-significant group differences | NR |
| Held et al., 2018 | Stroke | Reaching counts | NR | NR | Responsive |
| Lee et al., 2018 | Stroke | Physical activity energy expenditure | ICC = 0.953–0.980 | NR | NR |
| Lee et al., 2018 | Stroke | Goal-directed movement count | NR | C-statistic = 87.0% | NR |
| Klassen et al., 2017 | Stroke | Step count | NR | Slope = 0.99 vs SAM | NR |
| Bergamini et al., 2017 | Stroke | Acceleration attenuation | NR | NR | NR |
| Parkinson disease |  |  |  |  |  |
| De Pasquale et al., 2025 | PD | Stride length | NR | p < 0.001 | NR |
| Denk et al., 2022 | PD | Percentage time frozen | NR | Moderate correlations | NR |
| Lencioni et al., 2022 | PD | APA onset | NR | R = 0.99 | NR |
| Lee et al., 2021 | PD | Stride parameters | ICC = 0.64–0.87 | NR | NR |
| Albani et al., 2019 | PD | Gait | ICC = 0.61 | NR | NR |
| Botros et al., 2019 | PD | Daily wear time | NR | Mean wear time = 15 h/day | NR |
| Fiems et al., 2018 | PD | Sway balance score | ICC = 0.72–0.92 | NR | NR |
| Ozinga et al., 2017 | PD | Peak sway acceleration | NR | NR | NR |
| Bonora et al., 2015 | PD | ML trunk acceleration | NR | NR | NR |
| Mancini et al., 2015 | PD | Turn mean velocity | NR | Sensitive to PD impairment | NR |
| Spinal Cord Injury |  |  |  |  |  |
| Johnson et al., 2025 | SCI | Distance correlation features | NR | dCorr = 0.50–0.72 | NR |
| Giagiozis et al., 2025 | SCI | Daily energy expenditure | NR | r = .69 (SCIM), r = .63 (UEMS) | NR |
| Marco-Ahulló et al., 2021 | SCI | Energy expenditure | NR | r = 0.72 | NR |
| Schneider et al., 2018 | SCI | Activity counts | High reliability | NR | NR |
| Brogioli et al., 2017 | SCI | Upper-extremity activity count | NR | Correlated with independence | NR |
| Mixed neurological disorders |  |  |  |  |  |
| Caronni et al., 2024 | Mixed | Gait speed | NR | MDC = 0.13 m/s | NR |
| Caronni et al., 2023 | Mixed | Turning duration | NR | AUC = 0.69 | NR |
| Anastasi et al., 2019 | Mixed | Gait | NR | r = 0.84 | NR |
| Rauen et al., 2018 | Mixed | Position change detection | NR | 100% detection | NR |
| Cerebral palsy |  |  |  |  |  |
| Kim et al., 2022 | CP | Dynamic postural sway | κ = 0.973 | NR | NR |
| Gerber et al., 2021 | CP | Stride velocity | ICC up to 0.98 | NR | NR |
| Carcreff et al., 2019 | CP | Walking bouts | NR | Higher sensitivity/accuracy | NR |
| Multiple sclerosis |  |  |  |  |  |
| Block et al., 2022 | MS | Daily step count | NR | Associated with falls (p = .007) | NR |
| Tanoh et al., 2021 | MS | MSCopilot Walking score | NR | r = 0.65 | NR |
| Flachenecker et al., 2020 | MS | Stride length | NR | r = 0.955 | NR |
| Traumatic brain injury |  |  |  |  |  |
| Arippa et al., 2024 | TBI | Sway area | NR | p = .003 | NR |
| Spinocerebellar ataxia |  |  |  |  |  |
| Velázquez-Pérez et al., 2020 | SCA | Swing time variability | NR | Correlated with disease onset | NR |

ICC, intraclass correlation coefficient; AUC, area under the curve; MDC, minimal detectable change; MAE, mean absolute error; BBS, Berg Balance Scale; NR, not reported.
